# Identifying Risk and Protective Factors for Shift Work Sleep Disorder: Insights from UK Biobank Night Shift Workers

**DOI:** 10.3390/clockssleep7010014

**Published:** 2025-03-12

**Authors:** Jürgen Degenfellner, Susanne Strohmaier, Magdalena Zebrowska, Ingvild Saksvik-Lehouillier, Eva Schernhammer

**Affiliations:** 1 Department of Epidemiology, Center of Public Health, Medical University of Vienna, 1090 Vienna, Austria; degnl@zhaw.ch (J.D.);; 2 Institute of Physiotherapy, ZHAW School of Health Sciences, Katharina-Sulzer-Platz 9, 8400 Winterthur, Switzerland; 3 Department of Psychology, Norwegian University of Science and Technology, NO-7491 Trondheim, Norway; 4 Channing Division of Network Medicine, Department of Medicine, Brigham and Women’s Hospital and Harvard Medical School, Boston, MA 02115, USA; 5 Department of Epidemiology, Harvard T.H. Chan School of Public Health, Boston, MA 02115, USA

**Keywords:** shift work, sleep disorder, insomnia, shift work tolerance

## Abstract

Shift Work Sleep Disorder (SWSD) is a significant and highly prevalent condition affecting up to 48% of individuals with irregular work schedules. The diagnostic criteria for SWSD include persistent insomnia or sleepiness in relation to shift work, not attributable to other disorders or external factors. To explore risk factors of SWSD, we conducted a cross-sectional analysis among 10,787 night shift workers in the UK Biobank. To determine correlates of SWSD using multivariable-adjusted logistic regression models, a preselection of potential risk factors was made on the basis of previous literature. Self-identifying as ‘Asian or Asian British’ or ‘Black or Black British’ (compared to being ‘White’), male sex, and high scores on sociability, warmth and diligence were associated with lower odds for SWSD. We did not find significant associations of chronotype, frequency of alcohol intake, smoking, and time employed in current job with SWSD. These findings underscore the need for targeted interventions and workplace policies to mitigate the adverse effects of SWSD. Future research should aim to explore the mechanisms behind these associations and develop strategies to enhance shift work tolerance among night shift workers.

## 1. Introduction

Shift work is generally considered as work that takes place outside of a traditional 9-to-5 day-shift schedule, involving evening or night-shifts, early morning shifts or rotating shifts [1]. Shift Work Sleep Disorder (SWSD) is a condition that affects individuals who engage in work that involves regularly shifting from day to night shifts or working during unconventional hours. This condition is characterized by symptoms such as insomnia, sleep disturbances, and excessive sleepiness, which can negatively impact the quality of life and lead to a range of health issues. According to the International Classification of Sleep Disorders (ICSD 3 [2]), diagnosing SWSD requires identifying sleep disturbances, such as insomnia or excessive sleepiness that are caused by overlapping work and normal sleep schedules, persisting for over three months. Additionally, it is required that the sleep issues are not explained better by other causes. Lastly, at least 14 days of sleep log and actigraphy monitoring are necessary to document the condition.

The prevalence of SWSD is estimated to be around 26.5% according to a recent systematic review from 2021 by Pallesen et al. [3] with a 95% prediction interval ranging from 6% to 67%. Using a random population sample of three Australian cities, Di Milia et al. [4] estimated a prevalence of 32.1% among night workers and 10.1% in day workers. During the COVID-19 pandemic, the prevalence of shift work sleep disorder in shift nurses was estimated to be 48.5% [5] which was higher compared to estimates before the pandemic [6]. Reynolds et al. [7] reported an overall prevalence of 10.5% among workers on non-standard work schedules, with a range from 9.6% in early morning workers to 12.7% in rotating shift workers.

Since no objective diagnostic test is available for SWSD, questionnaires and clinical evaluation are used to diagnose the disorder. Flo et al. [8] used three questions to assess SWSD that adhered to the criteria listed in ICSD 2 [9]. Barger et al. [10] validated a 4-item screening questionnaire (starting with 26 questions) and reached adequate discriminatory properties (sensitivity = 0.74; specificity = 0.82) for their definition of SWSD. Taylor et al. [11] report reliability (Cronbach’s alpha = 0.94) and good convergent and divergent validity of the DSM 5 [12] informed Shift Work Disorder Index (SWDI).

Wang and colleagues [13] used a classification in which workers were considered to have SWSD if they experienced symptoms of insomnia and/or sleepiness and reported that their work schedule was the cause of these symptoms for at least three months. Waage et al. [14] used three questions based on the minimal criteria from ICSD 2 to assess SWSD status.

Drake et al. [15] report that individuals diagnosed with Shift Work Sleep Disorder (SWSD) are more likely to suffer from ulcers and are also at an elevated risk for accidents attributed to sleepiness. Additionally, they demonstrate increased rates of absenteeism from work and are more prone to experiencing depression. Importantly, the negative health impacts associated with SWSD were generally more severe than those observed in day workers who had comparable symptoms of sleep-related issues.

Given the high prevalence of SWSD and its implications for individuals’ health, well-being, and workplace performance, it is essential to identify the factors that contribute to the development and maintenance of this disorder. Understanding these factors could help inform targeted interventions and workplace policies aimed at mitigating the adverse effects of SWSD and improving overall shift work tolerance (SWT) which refers to an individual’s ability to adapt to and perform well in shift work schedules without experiencing significant health or sleep disturbances [16]. While previous studies have examined individual predictors of SWSD, our study is the first to simultaneously assess multiple factors associated with shift work tolerance, including demographic, behavioral, and psychological traits, within the UK Biobank.

## 2. Results

Table 1 presents the characteristics of the study participants, stratified by the duration of employment in their current job. The sample consisted of 10,787 ‘Usual’ or ‘Always’ night shift workers, with an average age of 51.1 (SD = 6.61) years and a range from 40 to 65 years. The sample was divided into rough quartiles based on the duration of employment in the current job: up to 5 years *n* = 3193), (5,11] years (*n* = 2316), (11,22] years (*n* = 2775), and (22,48] years (*n* = 2503). Overall, 24.5% (2643) of night shift workers suffered from insomnia disorder and hence were classified as having SWSD. Note, that the same number were classified as having frequent insomnia symptoms. The proportion of insomnia disorder was comparable across quartiles of work years, with slightly less cases in the up to 5-years category compared to the 22–48 years category (23% vs. 26%).

Most of the participants were male (62.2%) and identified as ‘White’ (86.6%). BMI was comparable in all groups with means ranging from 28.2 to 28.5 kg/m^2^ which can be classified as ‘overweight’ [17]. The distribution of chronotypes across quartiles was similar, whereas most participants reported to be ‘More an evening person than a morning person’ (ranging from 27.7–30.4%). 13.4% answered ‘Do not know’.

The proxies for personality traits and behavioral characteristics were stable across the quartiles of employment years. The mean of the diligence scale, for instance, shows a modest upward trend with longer years in the job in the first three quartiles. On the other hand, the mean curiosity scale shows a slight decrease in the same time spans.

52.7% of workers ‘never’ smoked. There is a trend of decreasing prevalence of current smokers with longer years in the job, while the number of people who have never smoked appears to increase. A large proportion of participants reported ‘Once or twice a week’ (29.6%) or ‘Three or four times a week’ (20.4%) for alcohol intake frequency. Larger differences in the distribution can be observed for ‘Special occasions only’ and ‘Three or four times a week’. Lastly, the frequency of alcohol intake also tends to increase with the number of years spent in the job, at least in the first three quartiles.

Table 2 compares the results of logistic regression models for both definitions of insomnia for both complete case analysis and imputation using mice. The main results are the ORs with imputed covariates for insomnia disorder (right column). The other three columns are given for comparison and reference. According to our statistical model, a wide range (3.3–69%) of probabilities for suffering from SWSD was predicted (see Figure S1).

Differences were observed across ethnicities. Individuals who identified as ‘Black or Black British’ exhibited lower odds (OR = 0.54; 95% CI: 0.42–0.69; *p* < 0.001) of insomnia symptoms and disorder compared to participants who identified as ‘White’. Note, that this estimate was very close to the complete case analysis (OR = 0.57; 95% CI: 0.38–0.82; *p* = 0.014) but with a narrower CI. Participants who identified as ‘Asian or Asian British’ had a similarly lower OR for insomnia disorder (0.45; 95% CI: 0.33–0.61; *p* < 0.001). Furthermore, individuals who identified as ‘Chinese’ or ‘Other ethnic group’ also had lower odds with ORs of 0.94 (95% CI: 0.53–1.68; *p* = 0.9) and 0.59 (95% CI: 0.42–0.84; *p* < 0.009), respectively, though the OR for ‘Chinese’ has a very wide CI.

Proxies for the big 5 personality traits were also associated with insomnia disorder. Higher levels of nervousness and curiosity were associated with higher odds of insomnia (1.18; 95% CI: 1.10–1.27; *p* < 0.001 and 1.06; 95% CI: 0.99–1.13, *p* = 0.2), while the latter did not show a clear distinction from the reference level. Higher scores on sociability (*p* < 0.001), warmth (*p* < 0.001), and diligence scales (*p* < 0.06) were associated with lower odds (ORs as low as 0.73 for warmth scale). The CIs for nervousness, sociability and warmth were relatively narrow, indicating a reasonable level of precision. Estimates for both curiosity and diligence had lower precision. To address distributional discrepancies between some of the scales, such as the nervousness and sociability scales, and the original distribution, we employed kNN imputation as an additional robustness check (Table S1). The results obtained through this imputation technique largely corroborated our initial findings. However, there were some noteworthy deviations: the odds ratio (OR) for the nervousness scale shifted to 1.07 with a 95% confidence interval of 1.00–1.13 (*p* = 0.1), and for the warmth scale, the OR adjusted to 0.90 with a 95% confidence interval of 0.84–0.95 (*p* = 0.002).

Age exhibited a positive relationship with insomnia disorder. The estimated odds ratio (OR) was 1.02 (95% CI: 1.02–1.03; *p* < 0.001), suggesting that with each year increase in age, the odds of experiencing insomnia increase by 2–3% which would mean 22–34% higher odds per ten years. The association of sex with insomnia disorder was more pronounced, with males showing lower odds (OR = 0.66; 95% CI: 0.60–0.73; *p* < 0.001) for insomnia disorder and (OR = 0.52; 95% CI: 0.46–0.58; *p* < 0.001) for frequent insomnia symptoms compared to females.

Estimates for chronotype were inconclusive for the levels ‘Do not know’ (0.93; 95% CI: 0.79–1.11; *p* = 0.5) and for ‘Definitely an evening person’ (1.07; 95% CI: 0.92–1.25; *p* = 0.5) compared to ‘Definitely a morning person’. The levels ‘More a morning than and evening person’ (0.85; 95% CI: 0.74–0.99; *p* = 0.07) and ‘More an evening person than a morning person’ (0.86; 95% CI: 0.75–0.99; *p* = 0.07) showed slightly lower odds for SWSD.

The estimated OR for alcohol intake showed comparatively large confidence intervals, e.g., the OR for daily alcohol intake was 1.12 but with low precision (95% CI: 0.91–1.39; *p* = 0.4). Participants with any missing values (respectively at least one missing big 5 scale) answered the question for alcohol intake frequency notable differently compared to the complete cases (Figure S2). Complete case analysis showed a larger OR of 1.67 (95% CI: 1.18, 2.37; *p* = 0.014) for daily alcohol intake compared to the imputed results.

BMI was positively associated with higher odds of insomnia disorder with an estimate of 1.02 (95% CI: 1.01–1.04; *p* = 0.003), which would mean a 10–48% increase in odds for insomnia disorder for a difference in 10 points on the BMI scale. Finally, the time employed in the current job showed no (OR = 1.00; 95% CI: 1.00–1.01; *p* = 0.5) association with insomnia disorder.

ORs for frequent insomnia symptoms where largely comparable with estimates for the proxies of the big 5 personality traits being more pronounced compared to insomnia disorder. Comparing the complete case and imputation-based results for each outcome there are some differences in estimates and precision, most notable for ethnicity, the personality traits and alcohol consumption. In sensitivity analyses we added all preselected covariables, one at a time, to the model. Results indicate that the estimates for the covariables remained largely unchanged (Table S2).

## 3. Discussion

We aimed [18] to explore the cross-sectional associations of a range of factors based on previous literature with the validated phenotype for insomnia disorder, which we used to define a proxy of Shift Work Sleep Disorder (SWSD)*,* among night shift workers in the UK Biobank based on an in-depth qualitative review [16] and a systematic review [18] up until 2011 to elucidate the architecture of shift work tolerance (SWT), which provided an overview of the research advances in the decade 2011–2021.

Overall, 24.5% (2643) of night shift workers in our sample were found to suffer from insomnia disorder. This figure aligns closely with the 26.5% prevalence of SWSD reported in a recent systematic review from 2021 by Pallesen et al. [3]. According to our statistical model, a wide range (3.3–69%) of probabilities for suffering from SWSD was predicted (Figure S1).

The age of in our sample of UKB participants was rather high (40–65 years), with a mean of 51.1 years (SD 6.61 years) which should be considered when interpreting the results for age and SWSD. Intuitively, it could be posited that with increasing age, workers might exhibit a greater tolerance for shift work. Factors contributing to this resilience may include diminished domestic obligations, particularly those associated with children, alongside an accumulation of general coping strategies. Additionally, it is generally observed that the need for sleep may decrease as individuals age [19]. Moreover, long-term shift workers inherently represent a ’survivor population’, having successfully adapted to the demands of such schedules over the years [20]. Contrast to this intuitive approach, all our models uniformly showed higher odds of SWSD for increasing age (1.02 per year; 95% CI: 1.02–1.03; *p* < 0.001), which would mean approximately 22% higher odds for SWSD per ten years. Not considering shift work, descriptive analysis by Hajak et al. [21] shows an increase in prevalence of severe insomnia with age from 1% in the 18–24 years category to 7% in the 55–64 years category. Flo et al. [8] report estimates for age of 1.01 (95% CI: 1.00–1.03, *p* < 0.05) for suffering from SWSD in a crude logistic regression respectively 1.05 (95% CI: 1.02–1.07) in an adjusted analyses. They used questions adhering to the symptoms/criteria listed in the ICSD 2 [9] and the mean age was lower compared to our sample (on average 32.8 years in the group without SWSD and 33.7 years in the SWSD group). Prevalence of SWSD was also considerably higher with 44.2%. The small differences in estimates for the regression coefficients for age might be attributable to the distinct operational definitions of SWSD and the use of alternative predictors in their model. For a sample of 700 nurses in Norway, Storemark et al. [22] report that age was associated negatively with sleep-related shift work tolerance for night-shifts. Booker et al. [23] report in a systematic review that age is positively associated with sleep problems in shift workers, including two longitudinal studies identifying older age as predictor for sleep-related impairment in the future [24,25]. In general, our results are in line with previous findings suggesting that prevalence of SWSD increases with age [26].

Regarding *sex*, being male was among the most pronounced associations with SWSD with an OR of 0.66 (95% CI: 0.60–0.73; *p* < 0.001). Proportions of SWSD for females and males were 29.8% and 21.1% respectively. Research has consistently demonstrated disparities between sex and/or genders in terms of sleep quality and efficiency [27]. Specifically, the risk of insomnia is greater in women than in men [28,29,30,31,32]. Generally, women often report having a lower quality of sleep based on their own assessments, even though this discrepancy isn’t backed up by objective sleep architecture data [33]. One factor that may contribute to this increased risk of sleep-related issues in women is the difference in circadian rhythms between the sexes. Women tend to have a circadian phase that is earlier in relation to their waking time, which results in an advanced circadian drive for alertness [34,35]. This phenomenon has been proposed as a reason for the more frequent occurrences of problems in sustaining sleep and waking up too early in the morning among women when compared to men [34,36]. Research also indicates that women report sleep difficulties more often than men [37,38]. Given the older age range of the UK Biobank population, menopause could be a contributing factor to the higher prevalence of sleep problems among females compared to males [39].

Contrary to our findings, Waage et al. [14] found the proportion of SWSD to be lower in women compared to men (7.7% vs. 11.5%, *n* = 1533) and report that having SWSD at baseline was positively associated with male gender. On the other hand, in the logistic regression model predicting SWSD at follow-up, the association with sex is unclear showing wide confidence intervals. Di Milia et al. [4] estimated higher odds ratios (1.78, 95% CI: 1.28–2.46; *p* = 0.001) for male sex. A study by Flo et al. [8] shows lower odds for females with regards to SWSD (0.57; 95% CI: 0.36–0.92, *p* < 0.05). In a systematic review by Booker et al. [23], eight of 14 studies indicated that sex did not impact sleep outcomes related to shift work, three studies found more insomnia, excessive sleepiness and poorer sleep quality in females, and three studies found poorer sleep outcomes in males. The discrepancies may stem from variations in sample size, number and type of covariables considered, age structure, sex imbalance (female dominated samples vs. 62% male in our sample), outcome definition (e.g., proxy vs. criteria from the ICSD 2), and the diversity of occupations within the study populations, such as a focus on nursing versus the inclusion of various job types (in our sample).

To the best of our knowledge, there are no studies reporting differences in prevalence of SWSD with respect to ethnicity [26]. In our analysis, ethnicity emerged as a pronounced predictor for SWSD. Individuals identifying as ‘Asian or Asian British’, ‘Black or Black British’ or ‘Other ethnic groups’ exhibited lower odds ratios (0.45; 95% CI: 0.33–0.61; *p* < 0.001 and 0.54; 95% CI: 0.42–0.69; *p* < 0.001 and 0.59; 95% CI: 0.42–0.84; *p* = 0.009), suggesting a decreased likelihood of SWSD compared to other ethnic groups (specifically to the reference group ‘White’). The prevalence of SWSD was notably lower in individuals identifying as ‘Asian or Asian British’, ‘Black or Black British’ or ‘Other ethnic groups’, with rates of 13.4%, 16.2%, and 17.5% respectively, as compared to those of White, Mixed or Chinese ethnicity, who exhibited higher prevalence of 25.6%, 28.0% and 25.4%. The evidence on disparities in insomnia, including difficulty initiating or maintaining sleep and early morning awakening, between Black and White adults is inconsistent [40]. Jean-Louis et al. [41] found that older African Americans reported fewer sleep-related complaints compared to European Americans. Similarly, Kaufmann et al. [42] observed higher insomnia scores among non-Hispanic Whites compared to non-Hispanic Blacks in a population aged 50 years and older. However, findings from the MESA study [43] suggest that Black adults reported experiencing insomnia more frequently than White adults. Ethnic differences in response bias may also play a role in the observed differences. One possible explanation is that older Black adults may have developed adaptive coping strategies to manage hardships like poverty, racism, and segregation, enabling them to reframe difficult life experiences more effectively over time [44,45]. More generally, there are ethnic differences in circadian rhythms and sleep [46]. Halder et al. have shown that African genetic ancestry is linked to differences in sleep structure [47] and that genetic ancestry may modulate sleep structure and the occurrence of sleep disorders, as observed in the São Paulo Epidemiologic Sleep Study (EPISONO) [48].

With regards to chronotype, the literature suggests that low scores of morningness are related to higher shift work tolerance [18]. Fischer et al. [49] described chronotypes using mid-point of sleep on weekends in the US using a large representative sample (*n* = 53,689). Figure 3b in the article depicts how chronotype (mid-point) changes with age for both sexes. Most of participants in our sample are between 40 and 60 years old and self-identified chronotypes were comparable when stratified by sex. ‘More a morning than and evening person’ (0.85; 95% CI: 0.74–0.99; *p* = 0.07) and ‘More an evening person than a morning person’ (0.86; 95% CI: 0.75–0.99; *p* = 0.07) slightly lowered the odds for SWSD. Survivorship bias may explain the discrepancies between our findings and previous literature. Given that individuals with a strong morning preference are less likely to tolerate shift work long-term, one would expect morning types to be underrepresented and later chronotypes to be overrepresented among current shift workers in the UK Biobank compared to the general population not engaged in shift work. This pattern is confirmed in our sample, where evening chronotypes are more prevalent among shift workers, while morning types are less common. For example, when looking at shift workers with ≥10 years of work experience, we observed that the proportion of definite evening types (15.5%) is nearly twice that of the general UK Biobank population (7.9%), while definite morning types are notably lower (18.6% vs. 24.3%). This further supports the idea that individuals with a natural evening preference are more likely to persist in shift work, whereas morning types are more likely to leave over time. Interestingly—also in the context regarding our finding on ethnicity—research has revealed notable circadian rhythm variations between racial and ethnic groups. Specifically, studies by the Eastman group in Chicago [50] found that African Americans have a shorter average circadian period than European-Americans by approximately 0.2 to 0.3 h, which is relatively large given the standard deviation of human circadian period is around 0.13 h. Additionally, analysis in the UK Biobank by Malone et al. [51] indicated that individuals identifying as black reported a twofold increase in the prevalence of short sleep duration (5 to 6 h) and were 1.4 times more inclined to describe themselves as morning or intermediate chronotypes compared to those identifying as white. For our sample (which is restricted to night shift workers), 21.4% and 28.6% describe themselves as ‘Definitely a morning person’ among people who identified as ‘Black or Black British’ respectively ‘Asian or Asian British’ compared to 18.6% for individuals who identified as ‘White’.

With respect to personality traits, according to Saksvik et al. [18], high scores on flexibility and low scores on languidity, low scores on neuroticism, high scores on extraversion and internal locus of control are related to higher shift work tolerance. Flexibility (of sleeping habits), languidity and internal locus of control were not available in the UK Biobank. Neuroticism and extraversion were included as proxies nervousness and sociability. High scores on the nervousness scale (1.18; 95% CI: 1.10–1.27; *p* < 0.001) and modestly the curiosity scale (openness) (1.06, 95% CI: 0.99–1.13; *p* = 0.2) increased the odds for SWSD, high scores on the sociability scale (0.74; 95% CI: 0.70–0.79; *p* < 0.001) (but also warmth) reduced the odds for SWSD. Both are in line with previous literature [18], Booker et al. [23] only mention hardiness and neuroticism, but extraversion did not show any consistencies in the results. One could hypothesize that individuals with high sociability and warmth may have stronger social networks, which can help mitigate the negative effects of shift work by providing emotional and practical support [52]. Hennig et al. [53] suggest caution in overemphasizing the connection between neuroticism (nervousness) and the ability to cope with shift work, pointing out individuals with high neuroticism scores tend to report complaints more frequently across various contexts, not exclusively within the realm of shift work. Similarly, Härmä [54] and Nachreiner [55] stress that personality traits are not reliable indicators of one’s capacity to endure shift work. They also note the challenges in ascertaining the causal relationship in this dynamic [18]. Unfortunately, hardiness, which is most consistently associated with Shift Work Sleep Disorder [16], was not available in the UKB. Hardiness seems to be negatively correlated with neuroticism (nervousness) and extraversion (sociability) [56], but this does unfortunately not guarantee that hardiness and SWSD are correlated [57,58].

Booker et al. [23] report that neither smoking nor alcohol consumption was consistently linked to sleep quality among shift workers. Alcohol use has been linked to the disruption of electrophysiologic sleep architecture and is associated with both insomnia and circadian abnormalities. Alcohol affects both quality and timing of sleep. Individuals consuming alcohol often experience a reduction in REM sleep, contributing to poor sleep quality [59]. Moreover, the relationship between alcohol consumption and sleep problems is bidirectional: frequent alcohol consumption can lead to insomnia, and pre-existing insomnia symptoms can drive individuals to consume alcohol as a sleep aid, perpetuating a cycle of poor sleep and continued alcohol use [60]. In our model, alcohol intake at ‘Special occasions only’ showed a modest reduction in the odds of developing SWSD (0.85; 95% CI: 0.70–1.03; *p* = 0.2). Conversely, ‘Daily or almost daily’ alcohol consumption resulted in somewhat higher risk of SWSD (1.12; 95% CI: 0.91–1.39; *p* = 0.4). Both ORs did not clearly show a difference from the reference level and the lack of temporal context regarding alcohol intake—specifically its proximity to sleep times—casts doubt on the practical significance of this finding compared to complete abstinence. Regarding tobacco use, both former (1.02; 95% CI: 0.91–1.14; *p* = 0.8) and ‘current’ smokers (1.08; 95% CI: 0.95–1.23; *p* = 0.4) exhibited a slightly, though not statistically significantly, higher SWSD risk. While there is a notable absence of specific studies directly investigating the relationship between smoking and SWSD, existing literature does offer insights into the interplay between smoking habits and sleep quality. There are discernible differences in both objective and subjective sleep parameters between smokers and non-smokers [61]. Smokers report more sleep problems than non-smokers [62]. Even secondhand smoking (SHS) is associated with higher odds for sleep disorder diagnosis and sleep disturbances [63,64] which might play a role for nonsmokers in a shift work settings because of evidence that shift workers smoke more often [65,66].

Night shift work, which can lead to poor sleep quality, disrupts the circadian rhythm. This disruption results in alterations to metabolic, inflammatory, neuroendocrine, and antioxidant biomarkers. Over time, these alterations could contribute to a higher incidence of obesity [67,68]. In general, shift work and specifically night shift work is a risk factor for obesity [69,70]. Di Milia et al. [4] report an association with SWSD of 1.04 (95% CI: 1.01–1.07; *p* = 0.001) in a crude logistic regression model respectively 1.03 (95% CI: 0.99–1.06; *p* = 0.121) in the adjusted logistic model for BMI which is comparable to our effect size of 1.02 (95% CI: 1.01–1.03; *p* = 0.003) but with lower precision probably due to the smaller sample size (*n* = 1163). They reported minimal BMI differences between participants with SWSD and those without (28.28 vs. 28.60), closely aligning with our findings of 28.32 for SWSD versus 28.75 for non-SWSD. This is also true for other studies [14,71].

The lack of association of time employed in the current job and SWSD may indicate that the development of insomnia disorder is less a function of job tenure (or not linearly dependent in the log-odds) and more likely influenced by other occupational factors not captured in this measure, such as shift patterns, job stress, or workplace environment. Alternatively, it might suggest that individual coping mechanisms or adaptations to the job role occur over time, mitigating potential cumulative effects of night shift work on sleep disturbance. It’s also plausible that the heterogeneity of job types within our sample obscures any nuanced relationship between time on the job and sleep quality. For instance, jobs with inherently disruptive schedules might show a stronger relationship with insomnia than those with regular hours, irrespective of job tenure. Another factor to consider is the potential impact of survivorship bias; specifically, night shift workers who may have developed SWSD due to prolonged night shift exposure could disproportionately leave their positions, thus being underrepresented in our cross-sectional sample. There is evidence that sleep problems become worse over time in shift workers in younger age groups (30–50 years) and less in older age groups (50 or more years, see Figure 2 in [72]), potentially increasing the pressure over time to leave shift work. This could help explain the estimate in our model.

Although our analysis focused on conventional predictors of SWSD, socioeconomic status (SES) indicators deserve further consideration. A secondary analysis (Table S3) demonstrated a significant relationship between deprivation index (a measure of deprivation in small areas), household income, education and the odds of insomnia disorder among current night shift workers. Ethnicity was added as a confounder. Among the seven individual deprivation scores (income, employment, health, education, housing, crime and living environment score) [73] health and housing scores showed associations with insomnia disorder whereas the income score was confounded by ethnicity. All estimated odds ratios were in the expected direction. This is also true for a wider body of research and specifically for ‘Sleep difficulty/insomnia’ (see Figure 2 in [74]). Lower SES is associated with more sleep problems [75,76,77], increased stress [78], poorer living conditions, and limited access to healthcare, which can exacerbate sleep disturbances. These challenges, combined with the irregular and demanding nature of night shift work, may make it more difficult for low-SES individuals to adapt to night shift work schedules, increasing their risk for developing SWSD.

In this study, we employed a rigorous approach by utilizing multiple imputation and k-Nearest-Neighbour imputation and a validated phenotype insomnia disorder. To the best of our knowledge, this was the first analysis to analyze the association between all Big 5 personality traits and SWSD. Also, to the best of our knowledge, this is the first study to consider the association between ethnicity and SWSD.

Our study’s limitations are important to be acknowledged. We used a cross-sectional design which limits our ability to establish temporal ordering between preselected covariables and the outcome proxies. The associations found might suffer from survivorship bias, since workers with a longer night shift work history might differ from the ones who quit in earlier stages. The proxy for SWSD allows for misclassification and results should therefore be interpreted with caution, e.g., it is not known if insomnia disorder was caused by night shift work. According to [79], “The condition can usually be diagnosed by history.” In our case, we do not have an onset time of SWSD or a detailed shift work history. In the UK Biobank, there is complete work history only for a rather small subset of participants. We did not include in the analysis the possibly further (night) shift work history (Figure S3). Another limitation is the reliance on a single self-reported question to determine insomnia disorder. Typically, insomnia is assessed using validated questionnaires such as the Insomnia Severity Index [80] or the Brief Insomnia Questionnaire [81], which provide a more comprehensive evaluation of insomnia symptoms and severity. The gold standard for diagnosing sleep disorders would be polysomnography (PSG). Its high cost and limited accessibility make it impractical for large-scale epidemiological studies. A promising alternative is polygraphy, which has been validated as a reliable diagnostic tool compared to full PSG [82].

Furthermore, the exact job type of UK Biobank participants is “regarded as restricted and will be supplied to researchers only when absolutely necessary” [83]. Big 5 personality scores had only integer levels from 0–5 but were treated as continuous variables. Also, model fit for the logistic regression model was modest but sufficient (Figure S4) and the reported estimates could suffer from omitted-variable bias. Furthermore, a binary classification into SWSD/no SWSD does not recognize the severity of the condition.

Additional research is essential to delineate the prevalence of Shift Work Sleep Disorder (SWSD) at global, national, and sector-specific levels. This will enable a more comprehensive understanding of both the individual and socioeconomic implications tied to this condition. Furthermore, it is crucial to identify both individual and organizational risk factors contributing to SWSD, as this will facilitate targeted identification and intervention for the populations most at risk.

## 4. Materials and Methods

### 4.1. Study Population

The UK Biobank is a population-based health research resource consisting of approximately 500,000 people, aged between 38 years and 73 years, who were recruited between the years 2006 and 2010 from across the UK [84]. Particularly focused on identifying determinants of human diseases in middle-aged and older individuals, UK Biobank provides a wealth of information (such as demographics, health status, lifestyle measures, cognitive testing, personality self-report, and physical and mental health measures) assessed via questionnaires and interviews; anthropometric measures, blood pressure readings and samples of blood, urine and saliva were also taken (data available at www.ukbiobank.ac.uk). A full description of the study design, participants and quality control (QC) methods have been described in detail previously [85]. UK Biobank received ethical approval from the Research Ethics Committee (REC reference for UK Biobank is 11/NW/0382).

### 4.2. Assessment of Shift Work Sleep Disorder (SWSD)

Insomnia disorder in the UK Biobank was defined based on self-report questions concerning trouble falling asleep and waking up in the middle of the night [86] ‘Usually’ (case) vs. ‘Sometimes/Never/rarely’ (control) and previously used by Hammerschlag et al. [87] in a genome-wide association study for insomnia complaints in the UKB. The study validated this phenotype using Insomnia Severity Index (ISI) and Pittsburgh Sleep Quality Index (PSQI) criteria and data from participants of the Netherlands Sleep Registry (NSR) in the same age range as UK Biobank participants (sensitivity 0.98, specificity 0.96, accuracy 0.97). We additionally defined frequent insomnia symptoms as having answered the question “Do you have trouble falling asleep at night or do you wake up in the middle of the night?” with ‘Usually’ (case) vs. ‘Never/rarely’ (control). This definition was used previously by Lane et al. [88]. This proxy overlaps with the primary outcome definition above since only the level ‘Sometimes’ was omitted from the original question for the definition of insomnia disorder. We have added this definition for sensitivity analysis and comparability purposes. SWSD was defined as insomnia disorder in night shift workers.

### 4.3. Analytic Sample

50,227 participants in the UKB answered the question “Does your work involve night shifts?” (Prefer not to answer/Never/Rarely/Sometimes/Usually/Always) during the initial assessment visit (2006–2010). We defined night shift workers as having answered ‘Usually’ or ‘Always’ (*n* = 11,084).

**Figure 1:**
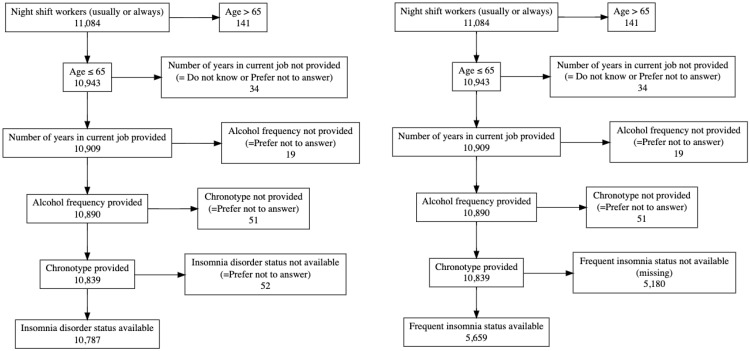
Exclusions for insomnia disorder (**left**) and frequent insomnia symptoms (**right**). Note that 4 participants who answered the question for night shift work were excluded due to withdrawal of consent before all analyses.

4 individuals, who withdrew consent, were excluded from the analysis beforehand. We restricted the age to less or equal 65 years (*n* = 141 > 65 years old were excluded). We excluded participants who answered the question “How many years have you worked in your current job? (If you have more than one job please answer this, and the following questions on work, for your MAIN job only)” with ‘Do not know’ or ‘Prefer not to answer’ (*n* = 34). Individuals who provided ‘Prefer not to answer’ as answer to the question “About how often do you drink alcohol?” were also excluded (*n* = 19). Likewise, participants who answered “Prefer not to answer” to their chronotype were excluded (*n* = 51). Participants without available insomnia disorder- or frequent insomnia status (yes/no) were excluded (*n* = 52 and 5180 participants), resulting in a total sample size of 10,787 for SWSD defined by insomnia disorder and 5659 for SWSD defined by frequent insomnia symptoms (Figure 1).

### 4.4. Covariables

Previously, we conducted an in-depth qualitative review [16] to elucidate the architecture of shift work tolerance (SWT), providing an overview of the research advances in the last decade (2011–2021) based on the systematic review of Saksvik et al. [18].

Specifically, hardiness, languidity, extraversion, negative affect, locus of control, rigidity, flexibility, neuroticism, resistance to change, self-esteem, age, gender, chronotype (morning type), and hormone levels were mentioned in this literature.

We searched for the previously identified variables associated with SWT in the UK Biobank.

Hardiness, languidity, locus of control, resistance to change and self-esteem were not directly available in the UKB. Extraversion and neuroticism are two of the Big 5 personality traits. We included the proxies to the Big five personality traits developed by Dahlén et al. [89] (see Table 1 of their article) creating nervousness-, sociability-, warmth-, diligence- and curiosity scales. The proxies have not been validated and should be interpreted with caution but were constructed in line with the methods used in other well-established short-form personality trait questionnaires [90]. The nervousness scale from Dahlén et al. and the neuroticism score, which both were directly available in the UKB, were strongly correlated (r = 0.86).

Age in years was included as integer. Biological sex was included with levels Female/Male. Note that this data field in the UKB may contain a mixture of the sex the NHS had recorded for the participant and self-reported sex [91]. Ethnicity was categorized with levels White/Mixed/Asian or Asian British/Black or Black British/Chinese/Other ethnic group and refers to a construct encompassing cultural characteristics including language, religion, dietary practices and nationality. It may also reflect common ancestry or geographic origin [92]. Chronotype was assessed by using a question derived from the Morningness-Eveningness Questionnaire [93,94]: “Do you consider yourself to be?” with answer options Definitely a morning person/Prefer not to answer/Do not know/More a morning than an evening person/More an evening person than a morning person/Definitely an evening person.

Other, potentially modifiable variables mentioned previously in the literature as being potentially linked with SWSD were alcohol and coffee consumption, smoking and body mass index (BMI) [18]. For both alcohol and coffee intake, participants were asked to provide an average considering the time span of the last year. Smoking status (Prefer not to answer/Never/Previous/Current) and alcohol intake frequency (Never/Prefer not to answer/Special occasions only/Once or twice a week/One to three times a week/Three or four times a week/Daily or almost daily) were also used as covariables in the models as well as BMI (measured in kg/m^2^).

‘Time on current job’ was enquired as ‘How many years have you worked in the current job?’. If participants answered ‘Less than a year’ the value was set to ‘0 years’.

All covariables considered in the analysis were assessed at baseline (around 2006–2010).

### 4.5. Statistical Analysis

#### 4.5.1. Missing Data

Covariables had missingness rates from 0 to 42.9% (Figure S5) depending on the definition of the proxies for the big 5 personality scales. In Dahlén et al. [89] these traits were defined analog to the neuroticism score in Pell et al. [95,96] where the neuroticism sum score was created by summing up the number of ‘yes’ answers across twelve questions into a single integer score for each participant while ignoring the answer options ‘Prefer not to answer’ and ‘Do not know’ (respectively adding 0). We assigned a missing value to the sum score if one of these two answer options were given in any of the items defining the scores. This results in high missingness rates (up to 42.9%) and demanded the use of imputation. As a general rule, it is advisable to apply imputation for a missingness rate exceeding 3% [97].

#### 4.5.2. Imputation to Handle Missing Values

All statistical analyses were performed with R [98] version 4.4.2. For multiple imputation [99] we used the R package mice [100] version 3.17.0. Items for the scales of the big 5 personality traits were imputed separately by passive imputation (see van Buuren and Groothuis-Oudshoorn [100] and van Buuren [99] for details and examples of the implementation and adaption of the predictor matrix). Polytomous regression imputation was used for unordered categorical data (more than two levels) and predictive mean matching (pmm) for numerical data. Distributions of imputed and original values were compared (see Tables S4 and S5 for an exemplary comparison). To assess the robustness of the results of multiple imputation with mice, we additionally used k-Nearest Neighbour Imputation (kNN) from the package VIM [101] for the main outcome which was not imputed.

#### 4.5.3. Outcome Modeling

Multivariate logistic regression models with all preselected covariables within the same model were used to calculate ORs and 95% CIs to describe the relationship between these descriptive covariables and our considered proxies for SWSD. To evaluate the respective contribution of each factor, in sensitivity analyses, we started with age and sex in the model and subsequently added each of the other covariables, one at a time, to the model (Table S1).

Results of multiple imputation were pooled [99] and the respective results were compared to complete case analyses as reference.

To account for multiple testing and control the false discovery rate in our analysis, we adjusted the *p*-values using the Benjamini-Hochberg procedure [102]. Due to the lack of specified a priori hypotheses to be tested and our study design, all *p*-values should be interpreted exploratively only.

## Figures and Tables

**Table 1:** Characteristics of 10,787 participants in the UK Biobank who reported to have currently worked night shifts at baseline.

	Years in Job				
	Up to 5 Years	>5 to 11 Years	>11 to 22 Years	>22 to 48 Years	Total
	(*n* = 3193)	(*n* = 2316)	(*n* = 2775)	(*n* = 2503)	(*n* = 10,787)
**Insomnia Disorder**					
No	2455 (76.9%)	1740 (75.1%)	2093 (75.4%)	1856 (74.2%)	8144 (75.5%)
Yes	738 (23.0%)	576 (24.9%)	682 (24.6%)	647 (25.8%)	2643 (24.5%)
**Frequent insomnia symptoms**					
No	898 (28.1%)	672 (29.1%)	791 (28.7%)	655 (26.2%)	3016 (28.0%)
Yes	738 (23.1%)	576 (25.1%)	682 (24.4%)	647 (26.0%)	2643 (24.5%)
**Age**					
Mean (SD)	50.3 (6.52)	51.2 (6.88)	50.4 (6.82)	52.8 (5.89)	51.1 (6.61)
Median [Min, Max]	50.0 [40.0, 65.0]	51.0 [40.0, 65.0]	50.0 [40.0, 65.0]	53.0 [40.0, 65.0]	51.0 [40.0, 65.0]
**Sex**					
Female	1228 (38.5%)	962 (41.5%)	1016 (36.6%)	868 (34.7%)	4074 (37.8%)
Male	1965 (61.5%)	1354 (58.5%)	1759 (63.4%)	1635 (65.3%)	6713 (62.2%)
**Ethnicity**					
White	2535 (79.4%)	1970 (85.1%)	2504 (90.2%)	2337 (93.4%)	9346 (86.6%)
Mixed	35 (1.1%)	20 (0.9%)	25 (0.9%)	16 (0.7%)	96 (0.9%)
Asian or Asian British	172 (5.4%)	94 (4.1%)	81 (2.9%)	51 (2.2%)	398 (3.7%)
Black or Black British	274 (8.6%)	144 (6.2%)	102 (3.7%)	56 (2.2%)	576 (5.3%)
Chinese	23 (0.7%)	10 (0.4%)	24 (0.9%)	12 (0.5%)	69 (0.6%)
Other ethnic group	144 (4.5%)	70 (3.0%)	30 (1.0%)	21 (0.8%)	265 (2.5%)
**BMI**					
Mean (SD)	28.5 (4.96)	28.5 (5.09)	28.2 (4.81)	28.5 (4.73)	28.4 (4.90)
Median [Min, Max]	27.8 [15.2, 52.0]	27.7 [15.5, 59.4]	27.7 [15.8, 56.9]	27.8 [16.5, 53.4]	27.8 [15.2, 59.4]
**Chronotype**					
Definitely a morning person	637 (19.9%)	425 (18.4%)	509 (18.3%)	471 (18.8%)	2042 (18.9%)
Do not know	445 (13.9%)	317 (13.7%)	372 (13.4%)	311 (12.4%)	1445 (13.4%)
More a morning than an evening person	746 (23.4%)	524 (22.6%)	608 (21.9%)	565 (22.6%)	2443 (22.6%)
More an evening person than a morning person	885 (27.7%)	689 (29.7%)	835 (30.1%)	762 (30.4%)	3171 (29.4%)
Definitely an evening person	454 (14.2%)	348 (15.0%)	427 (15.4%)	370 (14.8%)	1599 (14.8%)
**Nervousness scale**					
Mean (SD)	1.84 (1.49)	1.85 (1.53)	1.77 (1.50)	1.71 (1.45)	1.79 (1.49)
Median [Min, Max]	2.00 [0, 5.00]	2.00 [0, 5.00]	1.00 [0, 5.00]	2.00 [0, 5.00]	2.00 [0, 5.00]
**Sociability scale**					
Mean (SD)	2.97(0.8)	3.00 (0.82)	3.06 (0.77)	3.07 (0.80)	3.02 (0.80)
Median [Min, Max]	3.00 [1.00, 4.00]	3.00 [1.00, 4.00]	3.00 [1.00, 4.00]	3.00 [0, 4.00]	3.00 [0, 4.00]
**Warmth scale**					
Mean (SD)	3.51 (1.34)	3.47 (1.35)	3.58(1.34)	3.61 (1.29)	3.54 (1.33)
Median [Min, Max]	4.00 [0, 5.00]	4.00 [0, 5.00]	4.00 [0, 5.00]	4.00 [0, 5.00]	4.00 [0, 5.00]
**Diligence scale**					
Mean (SD)	2.26 (0.97)	2.30 (0.96)	2.35 (0.95)	2.42 (0.95)	2.33 (0.96)
Median [Min, Max]	2.00 [0, 4.00]	2.00 [0, 4.00]	2.00 [0, 4.00]	3.00 [0, 4.00]	2.00 [0, 4.00]
**Curiosity scale**					
Mean (SD)	2.24 (0.80)	2.23 (0.78)	2.22 (0.78)	2.15 (0.77)	2.21 (0.78)
Median [Min, Max]	2.00 [0, 4.00]	2.00 [0, 4.00]	2.00 [0, 4.00]	2.00 [0, 4.00]	2.00 [0, 4.00]
**Smoking status**					
Never	1617 (50.6%)	1194 (51.6%)	1508 (54.3%)	1369 (54.7%)	5688 (52.7%)
Previous	906 (28.4%)	723 (31.2%)	837 (30.2%)	773 (30.9%)	3239 (30.1%)
Current	658 (20.6%)	394 (17.0%)	419 (15.1%)	356 (14.2%)	1827(16.9%)
**Coffee intake**					
Mean (SD)	2.23 (2.61)	2.19 (2.47)	2.25 (2.41)	2.36 (2.56)	2.26 (2.52)
Median [Min, Max]	2.00 [0, 40.0]	2.00 [0, 20.0]	2.00 [0, 15.0]	2.00 [0, 24.0]	2.00 [0, 40.0]
**Alcohol intake frequency**					
Never	422 (13.2%)	268 (11.6%)	230 (8.3%)	146 (5.8%)	1066 (9.9%)
Special occasions only	529 (16.6%)	368 (15.9%)	354 (12.8%)	271 (10.8%)	1522 (14.1%)
Once or twice a week	932 (29.2%)	680 (29.4%)	828 (29.8%)	755 (30.2%)	3195 (29.6%)
One to three times a week	455 (14.3%)	300 (13.0%)	428 (15.4%)	352 (14.1%)	1535 (14.2%)
Three or four times a week	417(16.2%)	451 (19.5%)	603 (21.7%)	628 (25.1%)	2199 (20.4%)
Daily or almost daily	338 (10.6%)	249 (10.8%)	332 (12.0%)	351 (14.0%)	1270 (11.6%)

**Table 2:** Odds ratios (ORs) and 95% confidence intervals (CIs) for the associations between risk factors and frequent insomnia and insomnia disorder based on complete case analyses and pooled ORs based on multiple imputation.

	Frequent Insomnia Symptoms			Insomnia Disorder			Frequent Insomnia Symptoms			Insomnia Disorder		
	(Complete Case)			(Complete Case)			(Imputed)			(Imputed)		
	*n* = 2483			*n* = 4746			*n* = 5659			n = 10,787		
	OR	95% CI	*p*-Value *	OR	95% CI	*p*-Value	OR	95% CI	*p*-Value	OR	95% CI	*p*-Value
**Age at assessment center**	1.04	1.03, 1.05	<0.001	1.03	1.02, 1.04	<0.001	1.04	1.03, 1.04	<0.001	1.02	1.02, 1.03	<0.001
**Sex**												
Female												
Male	0.51	0.42, 0.61	<0.001	0.64	0.55, 0.75	<0.001	0.52	0.46, 0.58	<0.001	0.66	0.60, 0.73	<0.001
**Ethnicity**												
White												
Mixed	1.43	0.57, 3.60	0.6	1.20	0.57, 2.40	0.7	1.23	0.64, 2.20	0.7	0.95	0.59, 1.52	0.9
Asian or Asian British	0.68	0.35, 1.27	0.4	0.81	0.46, 1.37	0.7	0.40	0.28, 0.58	<0.001	0.45	0.33, 0.61	<0.001
Black or Black British	0.58	0.37, 0.92	0.052	0.57	0.38, 0.82	0.014	0.60	0.45, 0.81	0.004	0.54	0.42, 0.69	<0.001
Chinese	0.64	0.11, 3.40	0.7	0.65	0.15, 2.08	0.7	1.11	0.54, 2.28	0.8	0.94	0.53, 1.68	0.9
Other ethnic group	0.61	0.29, 1.21	0.3	0.75	0.40, 1.33	0.6	0.65	0.43, 0.97	0.078	0.59	0.42, 0.84	0.009
**Chronotype**												
Definitely a morning person												
Do not know	0.92	0.65, 1.30	0.7	0.94	0.70, 1.25	0.8	0.96	0.79, 1.18	0.8	0.93	0.79, 1.11	0.5
More a morning than an evening person	1.01	0.78, 1.32	>0.9	0.97	0.78, 1.21	0.9	0.97	0.81, 1.15	0.7	0.85	0.74, 0.99	0.070
More an evening person than a morning person	1.09	0.84, 1.41	0.6	0.93	0.76, 1.15	0.7	1.04	0.88, 1.23	0.7	0.86	0.75, 0.99	0.070
Definitely an evening person	1.00	0.74, 1.33	>0.9	1.07	0.84, 1.35	0.7	1.05	0.87, 1.27	0.8	1.07	0.92, 1.25	0.5
**Nervousness scale**	1.8	0.96, 1.22	0.4	1.05	0.95, 1.15	0.6	1.25	1.15, 1.37	<0.001	1.18	1.10, 1.27	<0.001
**Sociability scale**	0.57	0.50, 0.65	<0.001	0.69	0.62, 0.76	<0.001	0.66	0.61, 0.71	<0.001	0.74	0.70, 0.79	<0.001
**Warmth scale**	0.85	0.75, 0.97	0.034	0.91	0.82, 1.00	0.13	0.63	0.59, 0.67	<0.001	0.73	0.70, 0.77	<0.001
**Diligence scale**	0.82	0.73, 0.91	0.002	0.85	0.78, 0.93	0.002	0.91	0.84, 0.98	0.029	0.93	0.87, 0.99	0.060
**Curiosity scale**	0.98	0.87, 1.10	0.8	1.00	0.91, 1.10	0.9	1.12	1.03, 1.22	0.016	1.06	0.99, 1.13	0.2
**Alcohol intake frequency**												
Never												
Special occasions only	1.17	0.75, 1.81	0.6	1.15	0.81, 1.65	0.7	0.85	0.66, 1.08	0.6	0.85	0.70, 1.03	0.2
Once or twice a week	1.51	1.02, 2.25	0.092	1.38	1.01, 1.91	0.12	0.90	0.72, 1.13	0.7	0.86	0.71, 1.03	0.2
One to three times a week	1.25	0.81, 1.94	0.5	1.21	0.85, 1.73	0.6	0.91	0.71, 1.17	0.8	0.92	0.75, 1.13	0.5
Three or four times a week	1.68	1.12, 2.53	0.034	1.36	0.99, 1.90	0.14	0.99	0.78, 1.25	>0.9	0.86	0.71, 1.05	0.2
Daily or almost daily	1.82	1.18, 2.81	0.029	1.67	1.18, 2.37	0.014	1.22	0.94, 1.59	0.2	1.12	0.91, 1.39	0.4
**Smoking status**												
Never												
Previous	0.92	0.76, 1.13	0.6	0.99	0.84, 1.16	>0.9	1.04	0.91, 1.18	0.7	1.02	0.91, 1.14	0.8
Current	1.12	0.86, 1.45	0.6	1.10	0.90, 1.35	0.6	1.07	0.91, 1.26	0.8	1.08	0.95, 1.23	0.4
**BMI**	1.03	1.01, 1.05	0.025	1.02	1.01, 1.04	0.014	1.02	1.01, 1.04	<0.001	1.02	1.01, 1.03	0.003
**Time employed in current job**	1.01	1.00, 1.02	0.034	1.01	1.00, 1.01	0.042	1.00	1.00, 1.01	0.4	1.00	1.00, 1.01	0.5

Frequent insomnia symptoms were defined as having answered the question “Do you have trouble falling asleep at night or do you wake up in the middle of the night?” with ‘Usually’ (case) vs. ‘Never/rarely’ (control). Insomnia disorder was defined by answering to the question regarding trouble falling asleep and waking up in the middle of the night ‘Usually’ (case) vs. ‘Sometimes/Never/rarely’ (control). * False discovery rate correction for multiple testing.

## Data Availability

All R-Codes are available on request from the corresponding author. UKB data are available for bona fide researchers (https://www.ukbiobank.ac.uk/ accessed on 1 January 2023).

## Supplementary Materials

The following supporting information can be downloaded at: https://www.mdpi.com/article/10.3390/clockssleep7010014/s1. Figure S1. Histogram with density estimator and boxplot of predicted probabilities according to the logistic regression model (using kNN imputation before). The highest predicted probability (69%) was for a ‘White’ female at age 53, who was ‘Definitely an evening person’, drank alcohol ‘Daily or almost daily’, was 30 years in the current job, had a BMI of 32.6, never smoked, had a nervousness scale of 4, a sociability scale of 1, a warmth scale of 1, a diligence scale of 2 and a curiosity scale of 3. Interestingly, the status of insomnia disorder was ‘No’. The lowest probability (3.3%) was for an ‘Asian or Asian British’ male, who was 40 years old, ‘More a morning than an evening person’, drank alcohol at ‘Special occasions only’, was 7 years on the job, had a BMI of 26, never smoked, had a nervousness scale of 0, a sociability scale of 4, a warmth scale of 4, a diligence scale of 3 and a curiosity scale of 2. The status of insomnia disorder was ‘No’; Figure S2. Left: Answers to the question of alcohol frequency in night shift workers comparing complete observations in the big 5 personality traits with at least one missing. Right: Answers to the same question using complete cases with respect to the entire analysis compared with at least one variable missing; Figure S3. Estimation the proportion of night shift workers with insomnia disorder working multiple jobs at time of their first assessment by using employment history (begin and end years of jobs) and the date of the first visit at the assessment center. Among night shift workers who answered the question about their employment history, 1827 had one job at their first visit, 69 had two and four had three jobs. For respondents with three jobs, two out of four seemed correctly classified. The other two are undecidable (between one/two/three jobs) since the date for the first assessment center visit was given in full (YYY-MM-DD) while the start and end times for the individual jobs were given in years (YYYY). For respondents with two jobs, 30 were undecidable between one and two jobs. Adding/subtracting the 30 undecidable cases (which include 0–5 participants with Insomnia Disorder) with two jobs to the one-job category, would change the prevalence to 22% in the one-job category and to 13–26% in the two-job category. Subtracting the two undecidable cases from the three-job category would change the prevalence within this category to 0–50%. Hence, with the data provided, one cannot decide if multiple simultaneous jobs potentially influence the prevalence of Insomnia Disorder; Figure S4. Diagnostic plots for logistic regression of insomnia disorder after imputation with k-Nearest Neighbor Imputation (kNN) from the package VIM (1). The plot was produced with the R-package performance (2). McFadden’s R2 was 0.06 (3). Upper left: Some observations are outside the error bounds in the binned residual plot hinting towards a suboptimal model. Upper right: No influential observations. Lower left: Somewhat inflated variance, but acceptable; Figure S5. Missingness plot of 10,787 individuals in our study sample; Table S1. Logistic regression for insomnia disorder after imputation with k-Nearest Neighbour Imputation (kNN) from the package VIM (1); Table S2. Logistic regression models for insomnia disorder after imputation with k-Nearest Neighbour Imputation (kNN) from the package VIM (1); Table S3. Logistic regression for insomnia disorder during exploratory data analysis (EDA) estimating the association between Townsend deprivation index (4), household income, ethnicity, education and insomnia disorder in night shift workers; Table S4. Distribution comparison for imputed values for ethnicity. Imputation 1 is the original distribution, imputations 2–16 are the m = 15 imputations of multiple imputation created using the package mice (5). Original and imputed distributions are similar; Table S5. Distribution comparison for imputed values for sociability scale. Imputation 1 is the original distribution, imputations 2–16 are the m = 15 imputations of multiple imputation created using the package mice (5). Imputations are notably different from the original sociability scale distribution. References [103,104,105] are cited in the supplementary materials.
