# Supplementary material for: Identifying Risk and Protective Factors for Shift Work Sleep Disorder: Insights from UK Biobank Night Shift Workers
Source: Clocks Sleep. 2025 Mar 12;7(1):14. doi: 10.3390/clockssleep7010014 (PMC11941026; doi:10.3390/clockssleep7010014)
Supplement: Supplementary file 1 [file clockssleep-07-00014-s001.zip › clockssleep-3352966-supplementary.pdf]

Supplementary Information to:

Identifying Risk and Protective Factors for Shift Work Sleep Disorder: Insights from UK Biobank Night Shift Workers

Jürgen Degenfellner [1,2*], Susanne Strohmaier [1], Magdalena Zebrowska [1], Ingvild Saksvik-Lehouillier [3], Eva Schernhammer [1,4,5]

1 Department of Epidemiology, Center of Public Health, Medical University of Vienna, 1090 Vienna, Austria; susanne.strohmaier@meduniwien.ac.at (S.S.); magdalena.zebrowska@meduniwien.ac.at (M.Z.); eva.schernhammer@meduniwien.ac.at (E.S.)

2 Institute of Physiotherapy, ZHAW School of Health Sciences, Katharina-Sulzer-Platz 9, 8400 Winterthur, Switzerland

3 Department of Psychology, Norwegian University of Science and Technology, NO-7491 Trondheim, Norway; ingvild.saksvik.lehouillier@ntnu.no

4 Channing Division of Network Medicine, Department of Medicine, Brigham and Women's Hospital and Harvard Medical School, Boston, MA 02115, USA

5 Department of Epidemiology, Harvard T.H. Chan School of Public Health, Boston, MA 02115, USA

* Correspondence: eva.schernhammer@meduniwien.ac.at

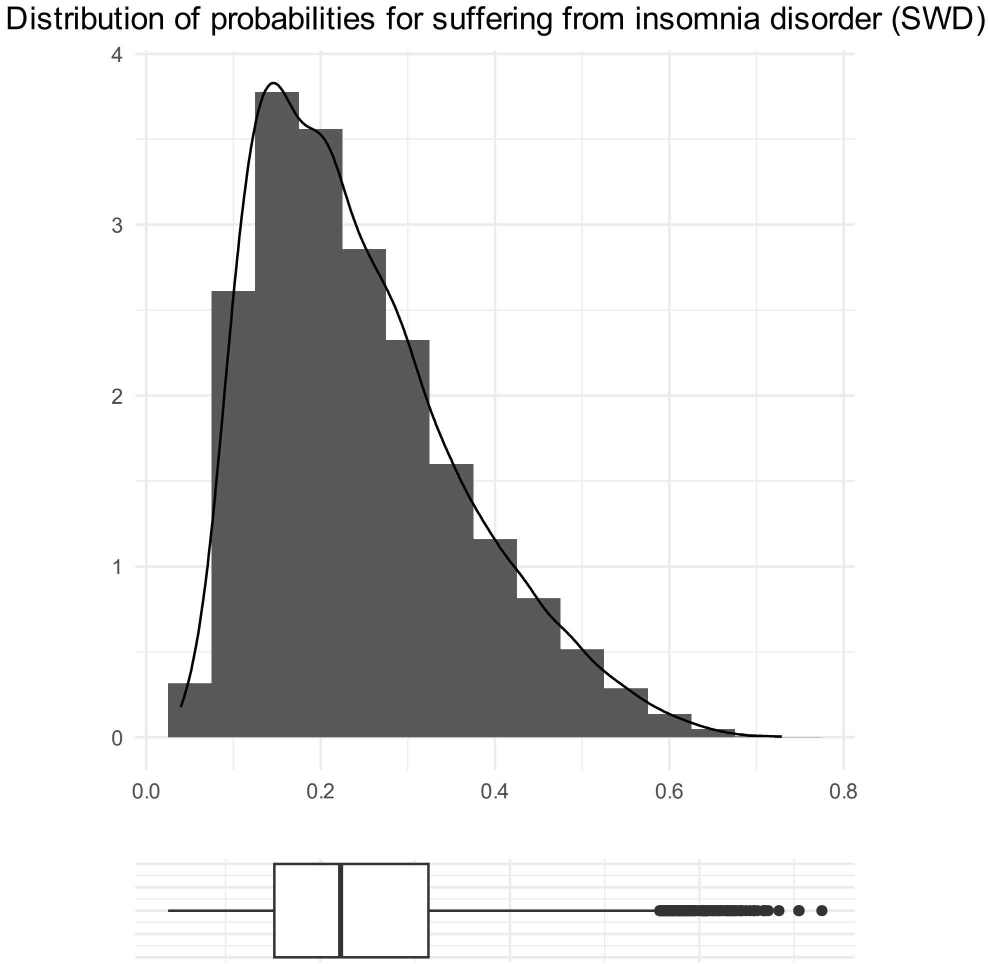

Figure S1. Histogram with density estimator and boxplot of predicted probabilities according to the logistic regression model (using kNN imputation before). The highest predicted probability (69%) was for a 'White' female at age 53, who was 'Definitely an evening person', drank alcohol 'Daily or almost daily', was 30 years in the current job, had a BMI of 32.6, never smoked, had a nervousness scale of 4, a sociability scale of 1, a warmth scale of 1, a diligence scale of 2 and a curiosity scale of 3. Interestingly, the status of *insomnia disorder* was 'No'.

The lowest probability (3.3%) was for an 'Asian or Asian British' male, who was 40 years old, 'More a morning than an evening person', drank alcohol at 'Special occasions only', was 7 years on the job, had a BMI of 26, never smoked, had a nervousness scale of 0, a sociability scale of 4, a warmth scale of 4, a diligence scale of 3 and a curiosity scale of 2. The status of *insomnia disorder* was 'No'.

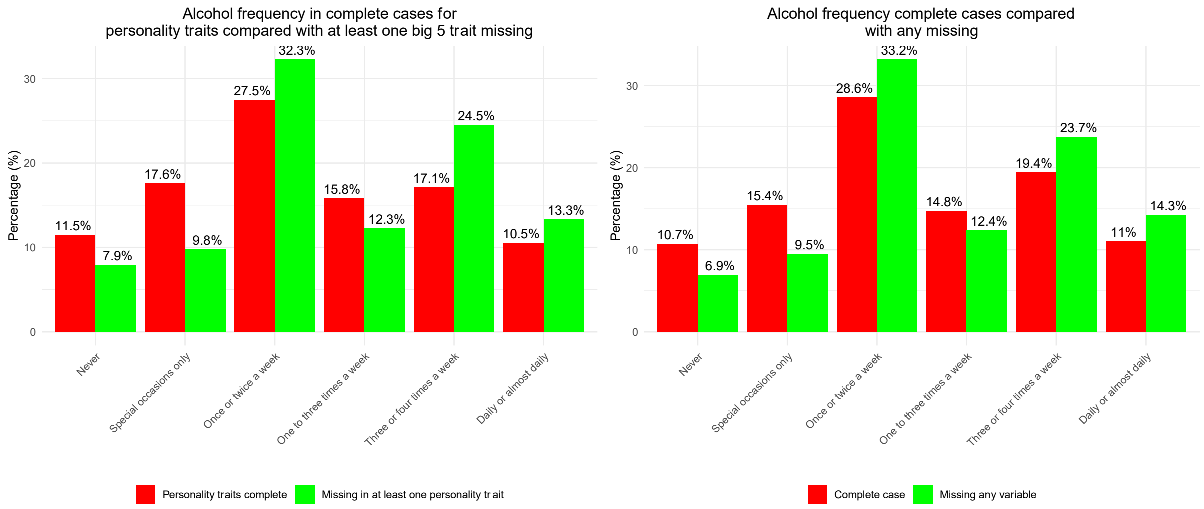

Figure S2. *Left*: Answers to the question of alcohol frequency in night shift workers comparing complete observations in the big 5 personality traits with at least one missing. *Right*: Answers to the same question using complete cases with respect to the entire analysis compared with at least one variable missing.

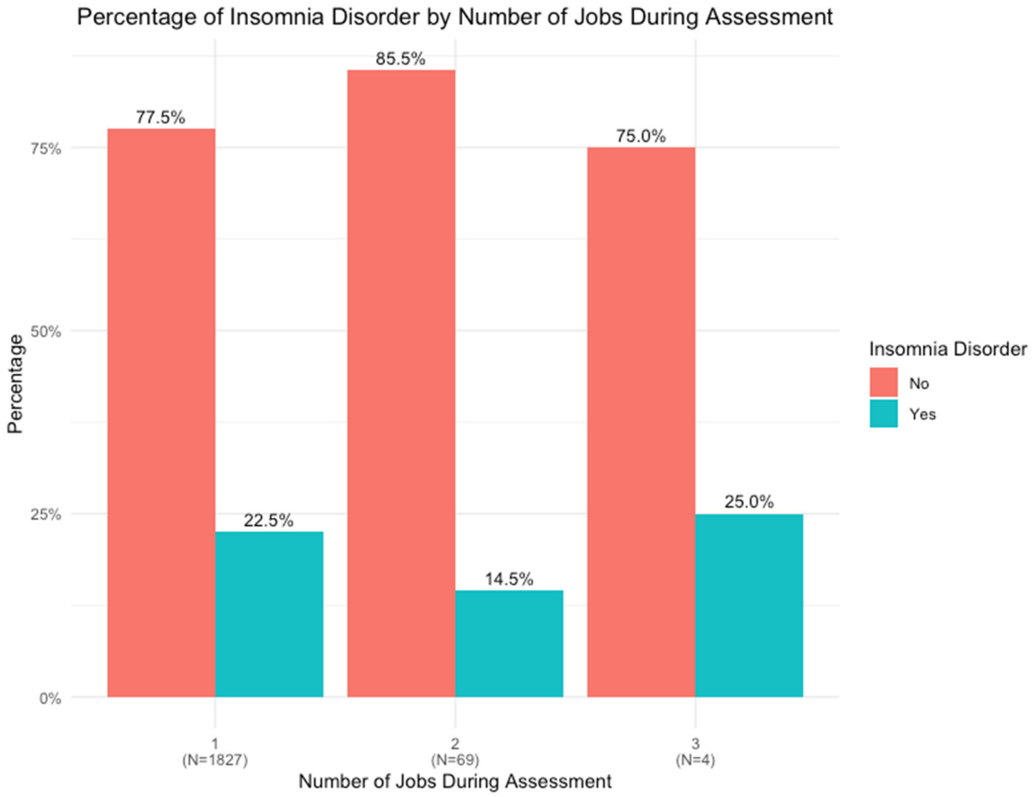

Figure S3. Estimation the proportion of night shift workers with insomnia disorder working multiple jobs at time of their first assessment by using employment history (begin and end years of jobs) and the date of the first visit at the assessment center. Among night shift workers who answered the question about their employment history, 1,827 had one job at their first visit, 69 had two and four had three jobs. For respondents with three jobs, two out of four seemed correctly classified. The other two are undecidable (between one/two/three jobs) since the date for the first assessment center visit was given in full (YYY-MM-DD) while the start and end times for the individual jobs were given in years (YYYY). For respondents with two jobs, 30 were undecidable between one and two jobs. Adding/subtracting the 30 undecidable cases (which include 0-5 participants with Insomnia Disorder) with two jobs to the one-job category, would change the prevalence to 22% in the one-job category and to 13-26% in the two-job category. Subtracting the two undecidable cases from the three-job category would change the prevalence within this category to 0-50%. Hence, with the data provided, one cannot decide if multiple simultaneous jobs potentially influence the prevalence of Insomnia Disorder.

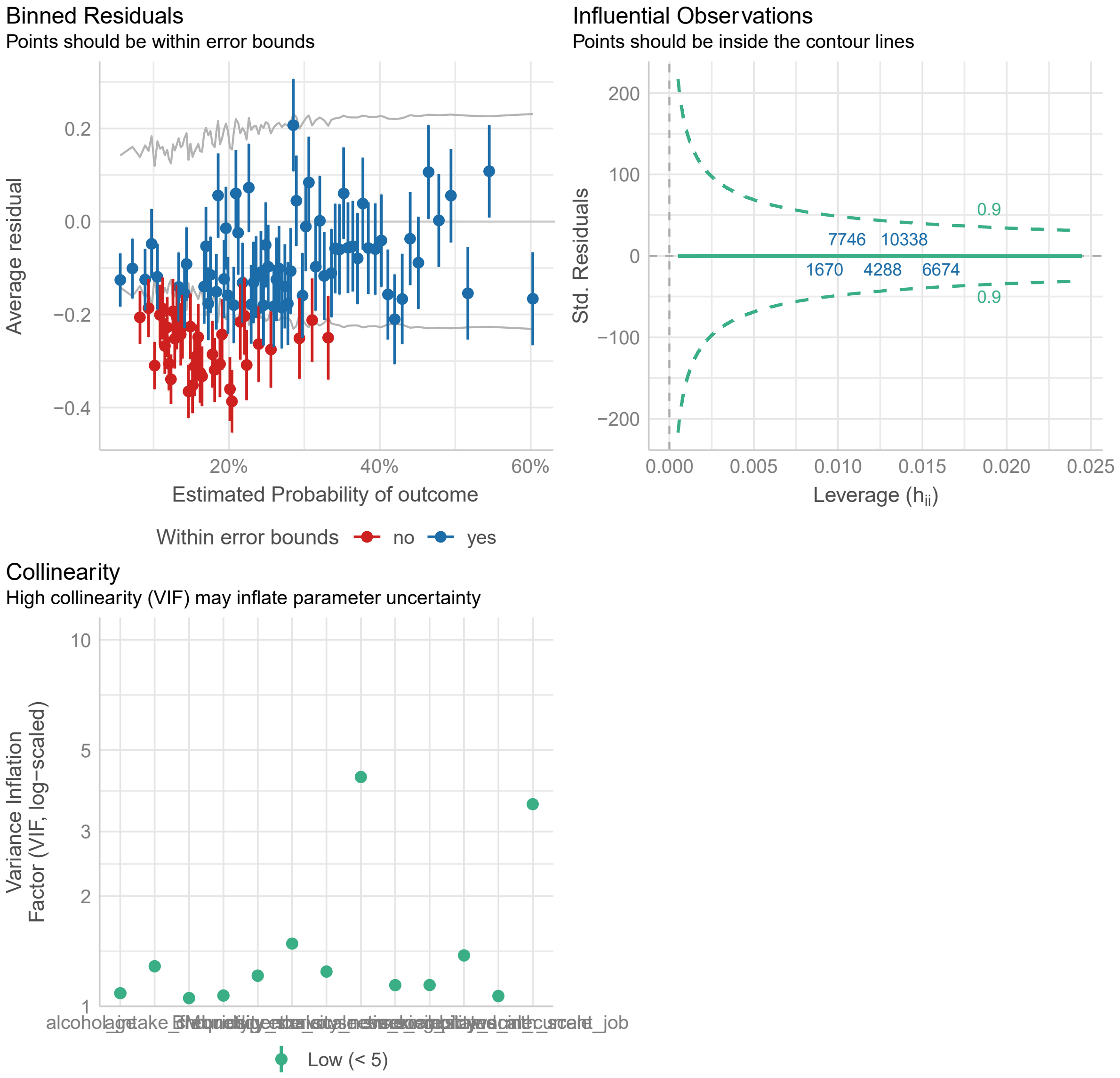

Figure S4. Diagnostic plots for logistic regression of *insomnia disorder* after imputation with k-Nearest Neighbor Imputation (kNN) from the package *VIM* (1). The plot was produced with the R-package *performance* (2). McFadden's R^2 was 0.06 (3). **Upper left**: Some observations are outside the error bounds in the binned residual plot hinting towards a suboptimal model. **Upper right**: No influential observations. **Lower left**: Somewhat inflated variance, but acceptable.

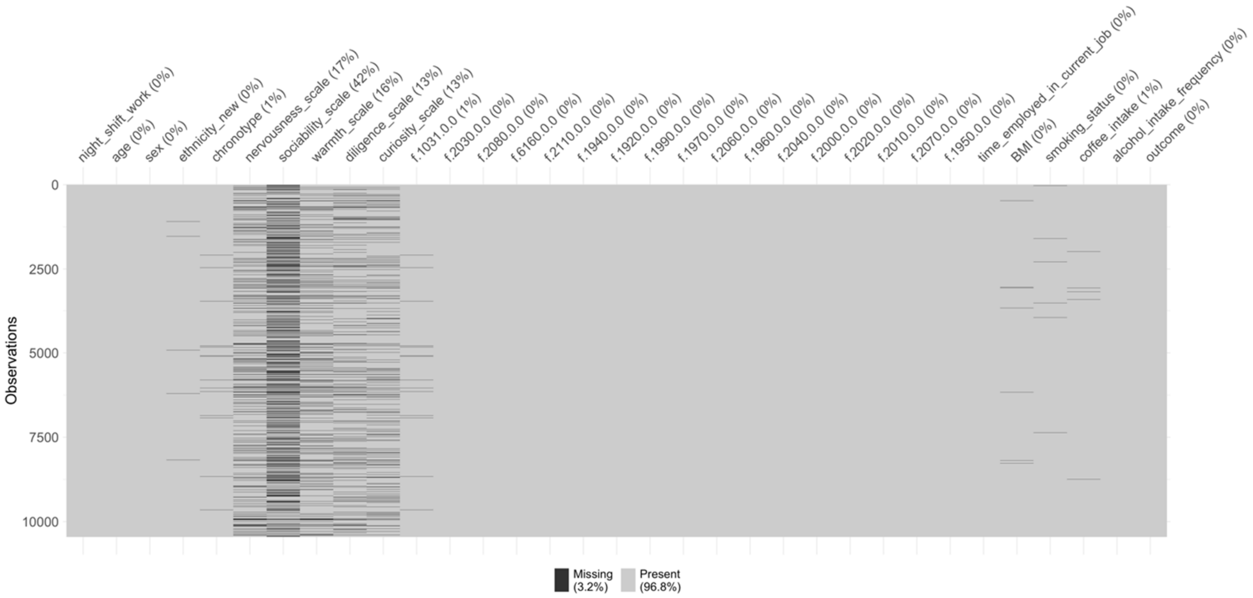

Figure S5. Missingness plot of 10,787 individuals in our study sample.

Table S1. Logistic regression for *insomnia disorder* after imputation with k-Nearest Neighbour Imputation (kNN) from the package *VIM* (1).

	OR	95% CI	q-value
**Age**	1.03	1.02, 1.03	<0.001
**Sex**			
Female			
Male	0.64	0.58, 0.71	<0.001
**Ethnicity**			
White			
Mixed	0.89	0.54, 1.41	0.7
Asian or Asian British	0.44	0.32, 0.59	<0.001
Black or Black British	0.47	0.37, 0.60	<0.001
Chinese	0.95	0.52, 1.67	0.8
Other ethnic group	0.53	0.38, 0.74	0.001
**Chronotype**			
Definitely a morning person			
Do not know	0.93	0.79, 1.10	0.5
More a morning than an evening person	0.84	0.73, 0.97	0.043
More an evening person than a morning person	0.82	0.72, 0.94	0.013
Definitely an evening person	1.03	0.89, 1.21	0.7
**Nervousness scale**	1.07	1.00, 1.13	0.1
**Sociability scale**	0.66	0.61, 0.70	<0.001
**Warmth scale**	0.90	0.84, 0.95	0.002
**Diligence scale**	0.91	0.86, 0.96	0.004
**Curiosity scale**	1.04	0.98, 1.11	0.3
**Alcohol intake frequency**			
Never			
Special occasions only	0.84	0.69, 1.02	0.14
Once or twice a week	0.86	0.72, 1.03	0.2
One to three times a week	0.91	0.74, 1.11	0.4
Three or four times a week	0.85	0.70, 1.03	0.2
Daily or almost daily	1.08	0.88, 1.32	0.6
**Smoking status**			
Never			
Previous	1.00	0.90, 1.12	>0.9
Current	1.08	0.95, 1.22	0.4
**BMI**	1.01	1.00, 1.02	0.019
**Time employed in current job**	1.00	1.00, 1.01	0.3

Table S2. Logistic regression models for *insomnia disorder* after imputation with k-Nearest Neighbour Imputation (kNN) from the package *VIM* (1).

	Model 1		Model 2		Model 3		Model 4		Model 5		Model 6		Model 7		Model 8	
	**OR**	**95% CI**	**OR**	**95% CI**	**OR**	**95% CI**	**OR**	**95% CI**	**OR**	**95% CI**	**OR**	**95% CI**	**OR**	**95% CI**	**OR**	**95% CI**
**Age at assessment center**	1.02	1.01, 1.03	1.02	1.01, 1.03	1.02	1.01, 1.03	1.03	1.02, 1.03	1.03	1.02, 1.03	1.03	1.02, 1.03	1.03	1.02, 1.03	1.03	1.02, 1.03
**Sex**																
Female																
Male	0.63	0.58, 0.69	0.62	0.57, 0.68	0.62	0.57, 0.68	0.66	0.60, 0.72	0.65	0.59, 0.72	0.65	0.59, 0.72	0.65	0.59, 0.72	0.65	0.59, 0.71
**Ethnicity**																
White																
Mixed			1.02	0.64, 1.59	1.01	0.63, 1.58	0.88	0.54, 1.40	0.86	0.53, 1.37	0.86	0.52, 1.37	0.86	0.52, 1.37	0.87	0.53, 1.38
Asian or Asian British			0.48	0.35, 0.63	0.47	0.35, 0.63	0.44	0.33, 0.59	0.43	0.31, 0.58	0.43	0.31, 0.58	0.44	0.32, 0.60	0.45	0.32, 0.60
Black or Black British			0.52	0.41, 0.65	0.53	0.42, 0.66	0.47	0.37, 0.60	0.46	0.36, 0.59	0.47	0.36, 0.59	0.46	0.36, 0.58	0.46	0.36, 0.59
Chinese			0.87	0.49, 1.48	0.86	0.48, 1.47	0.89	0.49, 1.55	0.89	0.49, 1.54	0.89	0.49, 1.55	0.94	0.52, 1.64	0.94	0.52, 1.64
Other ethnic group			0.59	0.42, 0.81	0.60	0.43, 0.82	0.53	0.38, 0.73	0.52	0.37, 0.72	0.52	0.37, 0.72	0.52	0.36, 0.72	0.52	0.37, 0.73
**Chronotype**																
Definitely a morning person																
Do not know					0.90	0.77, 1.06	0.91	0.77, 1.08	0.92	0.78, 1.08	0.92	0.78, 1.08	0.92	0.78, 1.08	0.92	0.78, 1.08
More a morning than an evening person					0.86	0.75, 0.98	0.83	0.72, 0.95	0.83	0.72, 0.96	0.83	0.72, 0.96	0.84	0.73, 0.96	0.84	0.73, 0.97
More an evening person than a morning person					0.90	0.79, 1.03	0.81	0.71, 0.93	0.81	0.71, 0.93	0.81	0.71, 0.93	0.81	0.71, 0.93	0.81	0.71, 0.93
Definitely an evening person					1.21	1.04, 1.40	1.01	0.87, 1.18	1.01	0.87, 1.18	1.01	0.86, 1.18	1.01	0.86, 1.17	1.00	0.86, 1.17
**Nervousness scale**							1.02	0.96, 1.09	1.03	0.97, 1.09	1.03	0.97, 1.09	1.02	0.96, 1.09	1.02	0.96, 1.09
**Sociability scale**							0.60	0.56, 0.65	0.61	0.56, 0.65	0.61	0.56, 0.65	0.61	0.57, 0.65	0.61	0.57, 0.65
**Warmth scale**							0.86	0.80, 0.91	0.86	0.81, 0.91	0.86	0.81, 0.91	0.85	0.80, 0.91	0.85	0.80, 0.91
**Diligence scale**							0.90	0.85, 0.96	0.90	0.85, 0.96	0.91	0.86, 0.96	0.91	0.86, 0.97	0.91	0.86, 0.97
**Curiosity scale**							1.07	1.00, 1.14	1.07	1.00, 1.14	1.07	1.00, 1.14	1.07	1.00, 1.14	1.07	1.00, 1.14
**Alcohol intake frequency**																
Never																
Special occasions only									0.85	0.70, 1.03	0.85	0.70, 1.03	0.84	0.70, 1.03	0.84	0.69, 1.03
Once or twice a week									0.87	0.72, 1.04	0.87	0.72, 1.04	0.88	0.73, 1.05	0.87	0.73, 1.05
One to three times a week									0.93	0.76, 1.13	0.93	0.76, 1.13	0.93	0.76, 1.14	0.93	0.76, 1.13
Three or four times a week									0.85	0.71, 1.03	0.85	0.71, 1.03	0.87	0.72, 1.05	0.86	0.71, 1.04
Daily or almost daily									1.06	0.86, 1.30	1.06	0.86, 1.30	1.08	0.88, 1.33	1.07	0.87, 1.32
**Smoking status**																
Previous											1.00	0.90, 1.12	1.00	0.89, 1.11	1.00	0.90, 1.12

Current	1.04	0.92, 1.19	1.06	0.93, 1.21	1.07	0.94, 1.22
**BMI**			1.01	1.00, 1.02	1.01	1.00, 1.02
**Time employed in current job**					1.00	1.00, 1.01

Table S3. Logistic regression for insomnia disorder during exploratory data analysis (EDA) estimating the association between Townsend deprivation index (4), household income, ethnicity, education and insomnia disorder in night shift workers.

	With ethnicity			Without ethnicity		
	OR	95% CI	p-value	OR	95% CI	p-value
**Deprivation Index (Scaled)**	1.06	1.00, 1.13	0.037	1.01	0.95, 1.06	0.8
**Income Group**						
Less than 18,000						
18,000 to 30,999	0.81	0.68, 0.96	0.014	0.84	0.71, 0.99	0.040
31,000 to 51,999	0.75	0.63, 0.89	0.001	0.80	0.68, 0.95	0.011
52,000 to 100,000	0.61	0.50, 0.74	<0.001	0.65	0.54, 0.80	<0.001
Greater than 100,000	0.79	0.53, 1.15	0.2	0.86	0.58, 1.25	0.4
**Ethnicity**						
White						
Mixed	0.90	0.51, 1.53	0.7			
Asian or Asian British	0.39	0.27, 0.56	<0.001			
Black or Black British	0.45	0.34, 0.59	<0.001			
Chinese	0.89	0.44, 1.65	0.7			
Other ethnic group	0.55	0.37, 0.78	0.001			
**Education**						
College or University degree						

Table S4. Distribution comparison for imputed values for ethnicity. Imputation 1 is the original distribution, imputations 2-16 are the m=15 imputations of multiple imputation created using the package *mice (5)*. Original and imputed distributions are similar.

**Imputation**	**White**	**Mixed**	**AsianOrAsianBritish**	**BlackOrBlackBritish**	**Chinese**	**Other ethnic group**
1	0,882	0,009	0,034	0,047	0,006	0,022
2	0,882	0,009	0,034	0,047	0,006	0,022
3	0,882	0,009	0,034	0,047	0,006	0,022
4	0,882	0,009	0,034	0,047	0,006	0,022
5	0,882	0,009	0,034	0,047	0,006	0,022
6	0,881	0,009	0,034	0,047	0,007	0,022
7	0,882	0,009	0,034	0,047	0,006	0,022
8	0,882	0,009	0,034	0,047	0,006	0,022
9	0,882	0,009	0,034	0,047	0,007	0,022
10	0,882	0,009	0,034	0,047	0,007	0,022
11	0,882	0,009	0,034	0,047	0,007	0,022
12	0,882	0,009	0,034	0,047	0,006	0,022
13	0,881	0,009	0,034	0,047	0,006	0,022
14	0,882	0,009	0,034	0,047	0,006	0,022
15	0,882	0,009	0,034	0,047	0,006	0,022
16	0,882	0,009	0,034	0,047	0,006	0,022

Table S5. Distribution comparison for imputed values for sociability scale. Imputation 1 is the original distribution, imputations 2-16 are the m=15 imputations of multiple imputation created using the package *mice* (5). Imputations are notably different from the original sociability scale distribution.

Imputation	Sociability scale				
	**0**	**1**	**2**	**3**	**4**
1	0	0,027	0,229	0,436	0,308
2	0,002	0,166	0,389	0,361	0,082
3	0,002	0,165	0,391	0,362	0,081
4	0,002	0,166	0,388	0,363	0,082
5	0,001	0,165	0,39	0,361	0,083
6	0,002	0,166	0,389	0,361	0,082

7	0,001	0,165	0,387	0,364	0,083
8	0,001	0,167	0,39	0,361	0,082
9	0,001	0,165	0,392	0,36	0,082
10	0,001	0,169	0,387	0,36	0,082
11	0,001	0,165	0,39	0,361	0,082
12	0,001	0,165	0,39	0,362	0,082
13	0,001	0,165	0,392	0,361	0,081
14	0,001	0,165	0,39	0,362	0,083
15	0,002	0,165	0,387	0,363	0,083
16	0,001	0,163	0,391	0,362	0,082

References

1. Kowarik A, Templ M. Imputation with the R Package VIM. Journal of Statistical Software. 2016;74(7):1 - 16.
2. Lüdecke D, Ben-Shachar MS, Patil I, Waggoner P, Makowski D. performance: An R package for assessment, comparison and testing of statistical models. Journal of Open Source Software. 2021;6(60).
3. Signorell A, Aho K, Alfons A, Anderegg N, Aragon T, Arppe A, et al. DescTools: tools for descriptive statistics. R package version 0.99. 26. Compr. R. Arch Netw. 2023:289-91.
4. Townsend P. Deprivation. Journal of Social Policy. 1987;16(2):125-46.
5. van Buuren S, Groothuis-Oudshoorn K. mice: Multivariate Imputation by Chained Equations in R. Journal of Statistical Software. 2011;45(3):1 - 67.
